# The Influence of Wet Granulation Parameters on the Compaction Behavior and Tablet Strength of a Hydralazine Powder Mixture

**DOI:** 10.3390/pharmaceutics15082148

**Published:** 2023-08-16

**Authors:** Oliver Macho, Ľudmila Gabrišová, Adam Guštafík, Kristian Jezso, Martin Juriga, Juraj Kabát, Jaroslav Blaško

**Affiliations:** 1 Institute of Process Engineering, Faculty of Mechanical Engineering, Slovak University of Technology in Bratislava, Námestie Slobody 17, 812 31 Bratislava, Slovakia; 2 Department of Analytical Chemistry, Faculty of Natural Sciences, Comenius University in Bratislava, Ilkovičova 6, 842 15 Bratislava, Slovakia; juraj.kabat@uniba.sk (J.K.); jaroslav.blasko@uniba.sk (J.B.)

**Keywords:** hydralazine, compaction, high-shear granulation, dynamic image analysis

## Abstract

The aim of this paper was to describe the influence of high-shear wet granulation process parameters on tablet tensile strength and compaction behavior of a powder mixture and granules containing hydralazine. The hydralazine powder mixture and eight types of granules were compacted into tablets and evaluated using the Heckel, Kawakita and Adams analyses. The granules were created using two types of granulation liquid (distilled water and aqueous solution of polyvinylpyrrolidone), at different impeller speeds (500 and 700 rpm) and with different wet massing times (without wet massing and for 2 min). Granulation resulted in improved compressibility, reduced dustiness and narrower particle-size distribution. A significant influence of wet massing time on parameters from the Kawakita and Adams analysis was found. Wet massing time had an equally significant effect on tablet tensile strength, regardless of the granulation liquid used. Granules formed with the same wet massing time showed the same trends in tabletability graphs. Tablets created using a single-tablet press (batch compaction) and an eccentric tablet press showed opposite values of tensile strength. Tablets from granules with a higher bulk density showed lower strength during batch compaction and, conversely, higher strength during eccentric tableting.

## 1. Introduction

Hydralazine is a drug that is commonly used to treat individuals with high blood pressure (hypertension). It belongs to the class of drugs known as vasodilators, which work by relaxing and widening blood vessels, thereby reducing the pressure inside them and allowing blood to flow more easily. Hydralazine is available in various forms, such as tablets [1], muco-adhesive tablets [2], capsules [3], and injectable solutions [4]. The parenteral route presents significant risks, including difficulty of drug removal or reversal, risks of infection, emboli and hypersensitivity reaction, or patient incompliance [5]. On the other hand, pharmaceutical tablets are the most common dosage form for oral administration, mainly because it is an easier way of drug intake by patients. Tablets are produced by the compaction process. The compaction process involves two stages; the reduction in volume of the powder, which will decrease the separation distance between particle surfaces, followed by the formation of inter-particle bonds [6]. A mixture of the drug and selected excipients together constitutes a formulation from which tablets with the required properties are produced. Singh et al. [7] in their work were concerned with the optimization of formulations of oral tablets with controlled release of hydralazine. However, it often happens that powder materials do not have good flow properties, are cohesive or very dusty, and it is necessary to modify their properties by the granulation process. Al-Suwayeh et al. [8] produced granules containing hydralazine using thermal granulation and then monitored the physical properties of the tablets created from them. The prepared tablets exhibited good and reliable sustained-release profiles of the drug. Mughal et al. [9] used the technique of extrusion and spheronization to produce hydralazine pellets. Vanitha et al. [10] and Acharya et al. [11] in their work dealt with the optimization and evaluation of floating tablets of hydralazine hydrochloride from granules created using wet granulation, designed to prolong the gastric residence time and to provide controlled release of the drug. Tablets must have a suitable mechanical strength to prevent chipping or breaking at manipulation, while ensuring adequate disintegration. Hegelstein et al. [12], in the case of compaction of tricalcium titrate, proved that the tensile strength value depends not only on the pressing parameters, but also on the properties of the pressed particles. Several authors have dealt with the issue of the relationship between tensile strength and compaction pressure [13,14,15]. The tensile strength of tablets also differs according to the type of press used [16]. The tensile strength of the tablet is thus a fundamental parameter to consider, and is therefore tested as standard during the manufacturing process [17]. In order for the tablets to have the required tensile strength, it is necessary to ensure their correct compressibility. One of the most frequently used methods for describing the compressibility of powder materials is the Heckel analysis [18,19,20,21]. Other frequently used analyses of material behavior during the compaction process include the Kawakita or Adams analysis [22,23,24]. The properties of the granules directly affect the properties of the tablets. Different parameters of the wet granulation process have different effects on the properties of the granules. Wang et al. [25] in their work dealt with the effect of the liquid-to-solid ratio, the type of wetting agent and the addition rate of the wetting agent on tabletability and on the properties of tablets made from granules using high-shear granulation. Wang et al. [26] investigated the effect of diluent types and granulation liquids on granule properties and tablet quality and the attribute transmission in the process. The present study deals with the modification of the properties of a dusty powder mixture containing hydralazine as an active substance, using the process of high-shear wet granulation. Until now, though, there have not been any published results where the wet granulation process of hydralazine was conducted not by sieving but rather in a high-shear granulator. The monitored parameters of the granulation process were the type of granulation liquid used, impeller speed and wet massing time. The formed granules were analyzed in terms of their properties and influence on tablet tensile strength. Compaction behavior was evaluated using the Heckel, Kawakita and Adams analysis. The tablets were produced using a single-tablet press (batch compaction), but also using an eccentric tablet press.

## 2. Materials and Methods

### 2.1. Experimental Material

A powder mixture of hydralazine as the active pharmaceutical ingredient (API) and excipients was kindly provided by the Department of Pharmaceutical Technology (Faculty of Pharmacy in Hradec Králové, Charles University, Prague, Czech Republic). The powder mixture consisted of 15% hydralazine (Teva, Opava, Czech Republic), 25% microcrystalline cellulose Avicel^®^ PH101 (DuPont, Cork Ireland) and 60% anhydrous lactose NF DT^®^ (Foremost, Rothschild, Los Angeles, CA, USA). All the measurements and manipulations with the powder, granules and tablets were carried out at a controlled ambient temperature of 25.0 ± 2.0 °C and a relative air humidity of 40.0 ± 8.0%, analyzed using an E5005 hygrometer (Emos, Prerov, Czech Republic).

### 2.2. Particle Size Analysis

The particle size and distribution of the powder mixture with hydralazine were analyzed using a Malvern Mastersizer 3000 laser diffraction analyzer (Malvern Panalytical, Malvern, UK), using the dry cell method. The particle refractive index and the particle absorption index were 1.56 and 0.01, respectively. Malvern software (v 3.62.) was used to determine d_10_, d_50_ and d_90_ values, corresponding to 10, 50 and 90% of a cumulative volume distribution curve, while the width of the particle distribution was expressed by the span parameter defined as d_90_ − d_10_/d_50_. The results were interpreted as an average value from three measurements.

### 2.3. Bulk, Tapped and True Density

The bulk and tapped density were analyzed using a TD1 tapped density tester (Sotax Pharmaceutical Testing, Prague, Czech Republic). The bulk density was calculated from the powder or granule mass and the initial volume of the sample. Tapped density was analyzed using Method 2 (250 taps/min from a height of 3 ± 0.2 mm), according to European Pharmacopoeia 10.0, 2.9.34 [27]. The final tapped volume was registered after 1250 taps. The final tapped density was calculated as a ratio of the powder mass and the tapped volume. The results were interpreted as an average value from three measurements. Compressibility of the powder mixture and granules was characterized using the compressibility index CI, calculated according to Equation (1).

(1) $$CI=\frac{100\;(V_{IN}-V_{1250})}{V_{IN}}$$

where *V_IN_* is the unsettled initial volume of the sample and *V*_1250_ is the final tapped volume. The true density of powder mixture samples was measured using an Ultrapyc 1200e helium pycnometer (Quantachrome Instruments, St. Albans, UK). A powder mixture sample with a volume of 10 cm^3^ was analyzed in a small sample cell. The results were interpreted as an average value from three measurements.

### 2.4. FT4 Compressibility

An FT4 powder rheometer (Freeman Technology, Tewkesbury, UK) was used to determine the compressibility of the powder mixture and granules as a function of applied normal stress. The batch of 80 g of powder or 85 g of granules, respectively, was filled into an assembly of two 85 mL measuring vessels. Before the compressibility measurement, three conditioning cycles using a 48 mm blade were carried out to remove entrapped air and ensure a homogeneously packed powder bed. The pretreated sample of tested material was split into a defined volume and the excess material was removed. The blade was replaced by a vented piston, which applied normal stress to the tested sample in a range of 0.5 to 15 kPa. Compressibility (*CPS*) from FT4 was calculated from the change in volume of the tested sample after the compression process, expressed as a percentage [28]. The results of the compressibility test were interpreted as an average value from three measurements. The FT4 compressibility measurement is not a direct indicator of flow properties, it is related to several operations such as storage in hoppers or the behavior of bulk materials during roller compaction [29].

### 2.5. Wet Granulation of the Powder Mixture

The process of wet granulation was carried out in a batch high-shear granulator of our own production [30], (WIPO (PCT) WO2017089976A1) [31]. Depending on the design, there are several types of high-shear granulators [32,33]. Our device has a volume of 6 L, a bottom-driven three-bladed impeller and a horizontally oriented five-blade chopper. The chopper speed was set at a constant 1000 rpm in all experiments. The batch of powder mixture was 200 g. The volume of added granulation liquid was the same, namely 78 mL, which represents a liquid-to-solid ratio of 0.39. The amount of liquid and also the granule moisture has a great influence on tablet tensile strength [34]. The experiments were performed with two types of granulation liquid. The first granulation liquid used was distilled water, the second a 3% aqueous solution of polyvinylpyrrolidone (PVP) K30 (BioChemica, Billingham, UK). The PVP solution was prepared before the granulation process, using a Stuart 162 magnetic stirrer (Stuart Equipment, Staffordshire, UK) at a temperature of 40 °C. The impeller speed was set to 500 rpm in half of the experiments and to 700 rpm in the others. Wet massing time defines the length of time the wet mixture is mixed in the granulator, without further addition of liquid. Half of the experiments took place with a wet massing time of 120 s, while the other half were carried out without wet massing (i.e., the granulation process was halted after adding the granulation liquid). Wet massing time can affect the uniformity of the granule composition [35]. The parameters of individual experiments can be found in Table 1. The formed granules were removed from the device, weighed and dried overnight at 40 °C.

### 2.6. Granule Size and Shape

The dried granules were analyzed using dynamic image analysis (DIA) on the PatAn 3D particle size and shape analyzer (Microtrac, Krefeld, Germany). The granules were characterized volumetrically, in agreement with ISO standards (13322-2, 9276-6) [36,37]. The mean granule size *d*_mean_ (mm) of area-equivalent diameter was selected as a characteristic dimension determining the size of individual samples of granules. The shape of individual granules was described using the shape coefficient of sphericity. The higher the sphericity value, the more the analyzed granule resembles a spherical particle. A sphericity value of 1 represents a perfectly spherical particle. The width of the granule size distribution was expressed in the same way by the span parameter as in laser diffraction analyses.

### 2.7. Angle of Repose

Angle of repose was analyzed in accordance with European Pharmacopoeia. The powder mixture and granules were poured through a funnel of 10 mm diameter onto a pad of 50 mm diameter until a cone was formed which did not change shape with continued pouring. Excess material fell over the edge of the pad. The created cones were photographed using a Xiaomi MiT 9 Pro camera (Xiomi Corporation, Beijing, China) and analyzed with ImageJ software v 1.53 (University of Wisconsin, Madison, WI, USA). The software allows the value of the angle to be measured directly, based on its delimitation by two straight lines. The results of the analysis of angle of repose were interpreted as an average value and standard deviation from three measurements.

### 2.8. Powder and Granule Compaction

Compaction of the powder mixture and its granules was performed on a Kistler NCFN60 electromechanical press (Kistler Eastern Europe, Prague, Czech Republic). The tablets were compacted in a steel jig formed of a die and a punch. The punch was of 13 mm diameter. Using a Nimbus NBL 254i analytical balance (Adam Equipment, Kingston, UK), the powder and granulated material were weighed to create tablets with a weight of 700 ± 5 mg. The tablets were compacted using three pressures, 50, 100 and 150 MPa, with a punch speed of 1 mm/s. Six flat-faced tablets were prepared from each sample, examined, and analyzed further.

### 2.9. Tensile Strength of Tablets

Tablet tensile strength, diameter and height were determined using an 8M hardness tester (Dr. Schleuniger Pharmatron, Solothurn, Switzerland) in accordance with the European Pharmacopoeia 10.0. The tensile strength *TS* (MPa) of six tablets was calculated according to Fell and Newton [38], using Equation (2).

(2) $$TS=2\times F_{B}/(\pi \times d\times h)$$

where *F*_B_ (N) is the radial force for breaking a tablet with diameter *d* (mm) and height *h* (mm).

### 2.10. Heckel Analysis

An in-die Heckel analysis [39] was used to characterize the compressibility of the powder and granulated material. The Heckel equation (Equation (3)) of pressure and relative density was created from the force-displacement dependences using known process parameters. This equation allows for the determination of the yield pressure needed to maintain plastic deformation. Relative density *D* is defined as the ratio between the density of the compact at pressure *P* and the true density of the solid particles obtained using a helium pycnometer.

(3) ln1/(1−D)=kP+A

where *k* is the slope of the straight-line portion of the Heckel plot and *A* is a constant characterizing particle rearrangement. Heckel yield pressure *P*_y_ (parameter of plasticity) was obtained as *P*_y_ = 1/*k*. The force and movement of the punch were measured using TraceControl V1.1.1.4.0 software for electromechanical presses.

### 2.11. Kawakita and Ludde Analysis

The second tool used to describe the compaction process was the Kawakita and Ludde equation (Equation (4)). The Kawakita and Ludde equation [40] was used to describe the relationship between volume reduction and applied pressure during the compaction process of powder and granular material.

(4) $$C=(h_{0}-h)/h_{0}=a*b*P/1+b*P$$

where *C* is the degree of compression (engineering strain), *h*_0_ is the initial bed height, and *h* is the height during load. The parameters *a* and *b*^−1^ correspond to the maximal engineering strain and the pressure required to reach *a*/2, and *P* is pressure. The reciprocal of *b* or *P*_k_ defines the pressure required to reduce the powder or granulate bed by 50%. The parameters were determined by linear regression from the linear form of the Kawakita and Ludde equation (Equation (5)).

(5) P/C=1/(ab)+P/a

### 2.12. Adams Analysis

The last tool for describing the compaction process is the Adams equation [41]. The Adams equation also describes the relationship between applied pressure and volume during compression, according to Equation (6).

(6) $$lnP=ln\frac{\tau_{0}}{\alpha }+\alpha *\varepsilon_{strain}+ln\left(1-\varepsilon^{-\alpha *\varepsilon_{strain}}\right)$$

where α is the friction coefficient,$\;\varepsilon_{strain}$ is the natural strain calculated as *ln* (*h*_0_/*h*), and *τ*_0_ is single granule strength derived from bed compaction, also known as the Adams parameter.

### 2.13. Tableting by Eccentric Press

The TDP 6s eccentric tablet press (LFA Machines, Bicester, UK) was used for producing multiple tablets and analyzing their uniformity of mass. The position of the upper and lower punches was adjusted according to the powder mixture in order to achieve a weight of the formed tablets of approximately 300–400 mg. The position was not further adjusted during granule pressing. The diameter of the punch was 10 mm. The tensile strength of the formed tablets was analyzed in the same way as in Section 2.9.

### 2.14. Uniformity of Mass

Ten tablets from the eccentric tableting machine were individually weighed using a Nimbus NBL 254i analytical balance (Adam Equipment, Kingston, UK). Uniformity of mass was determined as the standard deviation from the mean value. The results of weight variation between individual tablets were expressed in percentages.

## 3. Results and Discussion

### 3.1. Evaluation of Particle Size of Mixture

**Figure 1:**
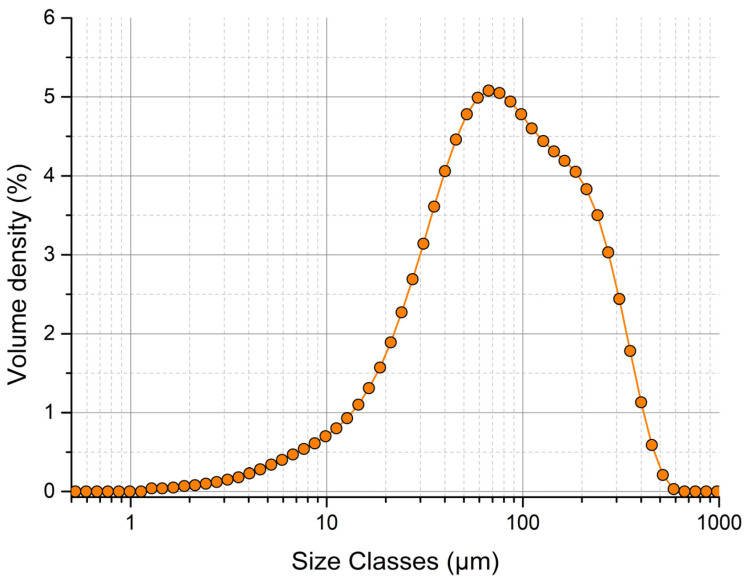
Particle-size distribution of experimental powder mixture analyzed by Mastersizer 3000.

The distribution curve of the experimental powder mixture with hydralazine as the active pharmaceutical ingredient was unimodal (Figure 1). Values *d*_10_ (21 μm), *d*_50_ (81.8 μm) and *d*_90_ (265 μm) were determined by the dry cell method. According to the tenth edition of the European Pharmacopoeia, Chapter 2.9.35 [27], particles with *d*_50_ < 125 μm are classified as very fine. The span parameter value was 2.98, which represents a material with a wide particle-size distribution. Based on laser diffraction results, it can be said that the powder mixture is made up of fine and very fine particles, which are problematic in terms of dustiness. This causes technological problems when this mixture is processed into the final tablet form.

### 3.2. Evaluation of Granule Size and Shape

**Figure 2:**
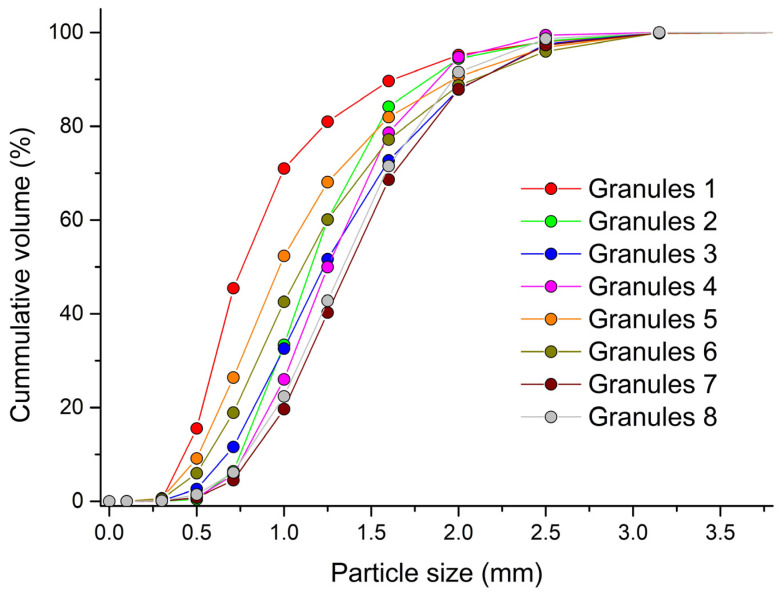
Particle-size distribution of the created granules analyzed by PartAn 3D.

The size distribution curve of the formed granules can be found in Figure 2. Along with the size of the granules, their sphericity and span were also analyzed. A summary of mean granule size, sphericity and span results can be found in Table 2. In almost all experiments with increasing wet massing time, the *d*_mean_ of formed granules increased. A longer wet massing time of the material enabled the continuation of the bonding, attrition and breakage mechanisms of the formed granules, which led to an increase in their size. The exception was granules made from distilled water at an impeller speed of 700 rpm. The binding mechanisms between the primary particles were not as strong when using water as a binder as when using a more-viscous PVP solution. This resulted in the smallest particle size in experiment G1 with the lowest impeller speed and without wet massing time. The use of a PVP solution as a granulation liquid resulted in the formation of granules with a larger *d*_mean_. The largest granules, with *d*_mean_ = 1.43 mm, were formed in experiment G7. According to the European Pharmacopoeia [27], particles with d_50_ > 355 μm are classified as coarse. Wet granulation thus eliminated the problem of dustiness of the powder mixture.

On the other hand, when PVP solutions were used, the formed granules had lower sphericity values. The highest value was recorded in experiment G1. Maroof et al. [42] classified particles with a sphericity value of 0.8–1.0 as high-spherical particles. All created granules can be included in this category. A longer wet massing time was also reflected in the width distribution of the formed granules. The granules that were created with a longer wet massing time were more consolidated and more uniform, which was reflected in the decrease in the span value. In all experiments with a wet massing time of 120 s, the values of span were less than 1.

### 3.3. Evaluation of Densities and Compressibility Index

The values from Table 2 show that the bulk density increases with a longer wet massing time. Similarly, with a wet massing time of 120 s, the value of bulk density increases with a faster impeller speed. The increase in bulk density values was caused by a more pronounced consolidation of granules, which were more dynamically stressed both by a longer mixing time and, at the same time, at higher impeller speeds. Higher impeller speeds cause a greater number of collisions between granules, which results in their densification. The powder mixture of hydralazine had a bulk density value of 0.654 g/mL. This value is higher than for granules created without wet massing, which were not sufficiently consolidated during the wetting phase. All the granules formed at a wet massing time of 120 s had bulk density values greater than the powder mixture. The highest values of bulk density were found for granules with PVP solution as a binder and a wet massing time of 120 s. The values of bulk density did not differ by more than 1% during repeated measurements; therefore, they are not listed in the table. Densification of the formed granules was also reflected in the *CI* values. More-consolidated granules achieved lower compressibility values in all the experiments. With a wet massing time of 120 s, the granules were less compressible than without wet massing. Granules that were created with a longer wet massing time had a lower span value and also a lower *CI* value. This phenomenon was caused by the fact that there was not such a wide distribution of particle sizes in the samples, and thus the space between large particles was not so intensively filled with small particles during dynamic loading. The powder mixture (span = 2.98) had a compressibility of 16.3%, which is, according to the European Pharmacopoeia [27], a value for fair flow properties. The mixture in the form of granules had a *CI* below 15%, which corresponds to good/free flow properties. The powder API had a value of *CI* = 25%, which represents a passable flow character. The smallest *CI* value was measured for G7 granules with the largest *d*_mean_ value.

### 3.4. Evaluation of Angle of Repose

Lower angle-of-repose (*AoR*) values, similar to the compressibility index, have powder or granular materials with better flow properties. The obtained *AoR* values can be found in Table 2. The table shows that the powder mixture of hydralazine had the highest *AoR* value (35.64 ± 0.26°). According to the European Pharmacopoeia, such a value is characteristic of materials that have flow properties at the interface between good and fair [27]. For comparison, pure API had a value of *AoR* = 55.31°, which represents the border between poor and very-poor flow properties. In accordance with the European Pharmacopoeia, all granules had an *AoR* < 35°, which represents good flow properties. G1 granules had the highest *AoR* value, which was due to the smallest *d*_mean_ size. For the other granule samples, no correlation was found between *AoR* and the size or shape of the granules formed. Deviations in *AoR* values were caused by interpolation of lines in the ImageJ software v 1.53, during repeated measurements.

### 3.5. FT4 Compressibility

Unlike the dynamic compressibility test performed on the TD1, the compressibility measurement with the FT4 is more of a static loading with a moving piston that applies a normal load to the material. The test results can be found in Table 2. The powder mixture of hydralazine had a value of *CPS* = 6.24 ± 0.24%. For comparison, pure API had a value of *CPS* = 11.44%. All the granules formed using water as granulation liquid had *CPS* values < 3%. Granules formed using PVP solution and without wet massing time had a *CPS* value of more than 3%. However, at a wet massing time of 120 s, samples G7 and G8 achieved the lowest compressibility. Compared to the powder mixture, a reduction in compressibility by more than 50% was achieved using the wet granulation process. When comparing *CI* and *CPS* measurement results, no direct dependence between these parameters was found. On the other hand, with PVP granules, both of these parameters were related to the span. More uniform granules created using PVP solution with a longer wet massing time also had lower values of the compressibility parameter in the FT4 static compressibility test. Highly compressible powder materials tend to cause storage problems (arching in the hopper) or uneven die filling during tableting. It can be said that the compressibility of the hydralazine powder mixture was modified by the wet granulation process.

### 3.6. Tabletability of Powder Mixtures and Granules

**Figure 3:**
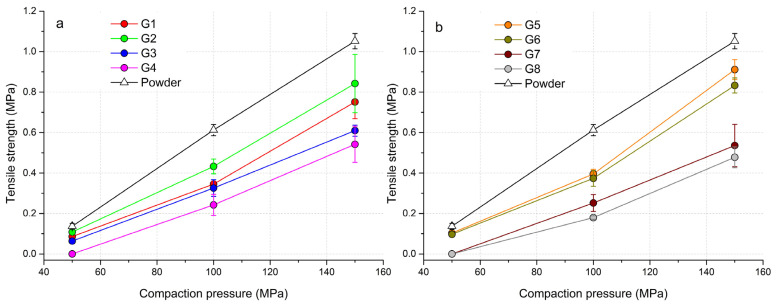
Tabletability profiles of powder mixtures and granules: (**a**) granules made with H_2_O, (**b**) granules made with 3% PVP solution.

With increasing compaction pressure, the tensile strength value (*TS*) of the formed tablets increased. Figure 3 shows that the highest strength was achieved in tablets made from a powder mixture of hydralazine. When the powder mixture and granules were pressed in the compaction pressure range of 50–150 MPa, no overcompaction phenomenon was observed. With G4 granules and a pressure of 50 MPa, it was not possible to create a solid tablet from the consolidated granulate that would show a *TS* value (Figure 3a). From the course of the curves, it can be seen that the tablets formed from the granulate with a wet massing time of 120 s had lower *TS* values. This phenomenon was caused by the fact that when the granulate was being pressed into the form of tablets, part of the energy had to be used for the fragmentation of the granules into particles and their rearrangement. A similar trend, but with a more pronounced separation of the curves, was also observed for tablets made from PVP solution as a granulation liquid (Figure 3b). With PVP granules, it was not possible to create a sufficiently strong tablet at the lowest compaction pressure. Conversely, granules formed from PVP without wet massing time and at a pressure of 150 MPa achieved the highest *TS* values of all experiments (*TS*_G5_ = 0.911 ± 0.049 MPa, *TS*_G6_ = 0.833 ± 0.037 MPa). In the case of granules without wet massing time, regardless of the granulation liquid used, a sharp increase in *TS* values occurred after the compaction pressure of 100 MPa was exceeded. Granules from distilled water created at a wet massing time of 120 s achieved higher values than granules from PVP at the highest compaction pressure (*TS*_G3_ = 0.609 ± 0.027 MPa, *TS*_G4_ = 0.542 ± 0.089 MPa). The longer wet massing time, which was also reflected in the span values of the formed granules, also partially affected the strength of the formed tablets. The wider distribution of particle sizes in the samples enabled the space between large particles to be filled up with small particles. There was an increase in density during their rearrangement in the die, resulting in stronger tablets. Not so much energy had to be spent on fragmentation and rearrangement of particles, but energy was spent on plastic deformation. Based on these findings, it can be said that the granulation wet massing time has a significant effect on the strength of the tablet, even with different types of granulation liquid. A higher compaction pressure would have to be used if tablets from granules with a *TS* of more than 1 MPa needed to be created.

### 3.7. Evaluation of Compaction Processes

**Figure 4:**
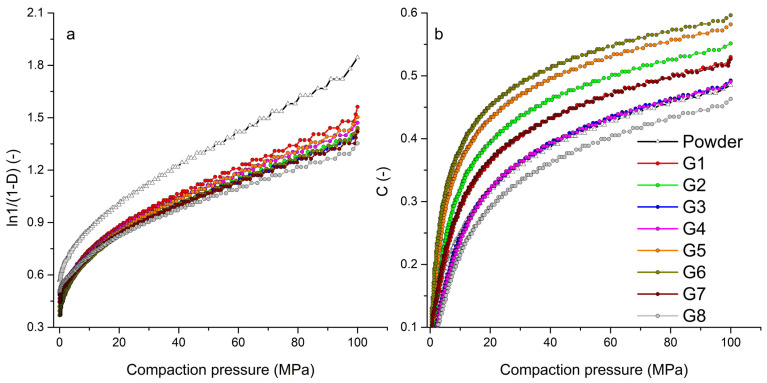
Graphical representation of tableting process analyses: (**a**) Heckel analysis, (**b**) Kawakita analysis.

Figure 4a shows the Heckel graph, the dependence of the logarithm of the relative density on pressure, for the powder mixture of hydralazine and individual granules. The graph shows a significantly different curve trend for the powder mixture, which stems from its different behavior during compaction. The steep curve of the powder mixture indicates a different transition of the compacted material into the area of plastic deformation. From the point of view of the Kawakita graph (Figure 4b), such a significant difference between the compacted materials was not recorded. The powder mixture curve was located near the G3 and G4 curves. Similar to the Kawakita analysis, there was no significant difference between the curves of powder mixture and granules obtained from the graph of logarithm pressure versus natural strain in the Adams analysis.

**Figure 5:**
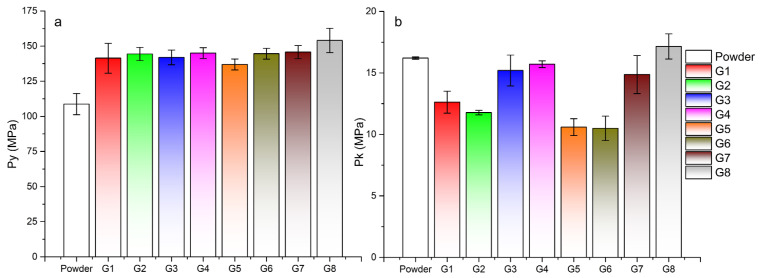
Parameters obtained from the analysis of the compaction process: (**a**) Heckel analysis—*P*_y_ parameter of plasticity, (**b**) Kawakita analysis—*P*_k_ parameter of 50% volume reduction.

Data from the force-displacement dependences were analyzed using the in-die method. Tablets created at a compaction pressure of 100 MPa were analyzed. The linear range for determining the parameters of Heckel and Kawakita was 20–100 MPa from the range of compaction pressure. In the Adams analysis, a natural strain value of 0.3–1 was considered as a linear region. In Table 3, there is a summary of all parameters of the compaction process. When evaluating the Heckel parameter of plasticity *P*_y_, the lowest values were obtained for the powder mixture. The higher the value of *P*_y_, the higher the pressure required to bring the compacted material into the area of plastic deformation. The lowest value of *P*_y_ (108.67 ± 7.56 MPa) in the powder mixture resulted in the fastest transition to the area of plastic deformation, which resulted in the creation of the strongest tablets. This value was significantly lower than the compaction of granule samples (Figure 5a). During the granule compaction process, part of the energy supplied by pressing was used for granule fragmentation and their subsequent rearrangement. Granules from sample G8 had the highest *P*_y_ value (153.96 ± 8.64 MPa), the tablets of which had the lowest strength of all (*TS*_G8_ = 0.18 ± 0.01 MPa at 100 MPa compaction pressure). In the case of granules made from distilled water, the average values of *P*_y_ were lower at lower impeller speeds of the granulator, as in the case of granules made from PVP, but higher values were achieved in the granules made with a longer wet massing time.

The most significant difference in the compaction of granules created according to different process parameters was found in the Kawakita and Adams analysis. Figure 5b shows a bar graph of *P*_k_ parameter values for tablets from a powder mixture and individual granules. *P*_k_ defines the pressure required to reduce the powder mixture or granulate bed by 50%. It is clear from the graph that more-consolidated granules created with a longer wet massing time needed more pressure to reduce their volume by half. High values of the *P*_k_ parameter were also achieved by the powder mixture with a wide distribution of particle sizes. Small particles in the mixture could fill the gaps between larger particles, which resulted in an increase in powder bed density and its reduced compressibility. The lowest *P*_k_ values were obtained by granules G5 and G6, which also had the highest values of the *CPS* compressibility parameter from the powder rheometer. For granules created without wet massing time and with an increase in the granulator impeller speed, the value of *P*_k_ decreased slightly; conversely, with a wet massing time of 120 s, it increased. The granules created with a longer wet massing time were more consolidated and thus had a higher density. Granules G8 with the highest bulk density (0.693 g/mL) had, in addition to the highest value of *P*_y_, also the highest value of the parameter *P*_k_ = 17.15 ± 1.02 MPa. It can therefore be said that the granules created at higher impeller speed and with longer wet massing time had the highest strength. The data from the Adams analysis displayed the same trend, but with shifted τ_0_ values. A similar correlation between τ_0_ and *P*_k_ when mixtures are compacted with paracetamol, can be found in the work of Patel et al. [43]. G8 granules had the highest single granule strength τ_0_ = 13.45 ± 1.62 MPa. Similar to the Kawakita analysis (Figure 5b), granule samples with the same wet massing time formed pairs with approximately the same values. It can be said that the influence of wet massing time on the compaction process was greater than the influence of impeller speed or type of granulation liquid used.

### 3.8. Evaluation of Eccentric Tableting

**Figure 6:**
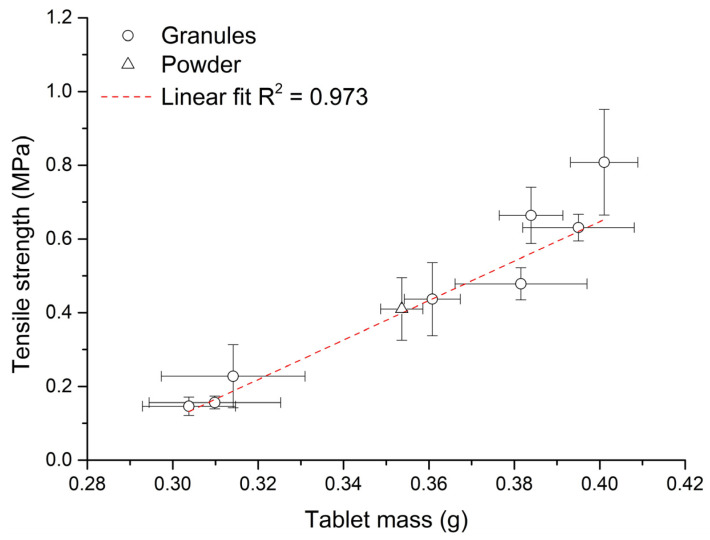
Dependence between tablet mass and their tensile strength during the eccentric tableting process.

Figure 6 shows the dependence between tablet mass and their tensile strength during eccentric tableting. The graph shows a strong linear dependence between these parameters (R^2^ = 0.973). As during eccentric tableting constant heights of the punch stroke were set on the press (constant volume of the die), it can be said that the mass of compacted material has a significant influence on the strength of the formed tablet. With the same pressure, a denser powder bed is compressed and the resulting tablet is therefore stronger. The values of uniformity of mass (Table 3) of the formed tablets were the lowest, with eccentric compaction of powder mixture (UoM = 1.39%). When pressing the powder mixture, small particles were compressed, which resulted in better repeatability, unlike granules with a size exceeding 1 mm. No direct relationship between UoM and *d*_mean_, or UoM and span was found during granule compaction. Except for the tablets from G2 granules, the deviation between the weights of the formed tablets was not more than 5%. Tablets formed from G1, G3 and G8 granules had differences in weight between them of less than 2%.

**Figure 7:**
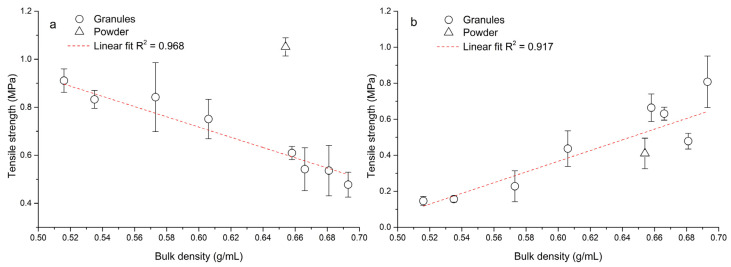
Dependence between bulk density and tablet tensile strength: (**a**) single-tablet compaction at 150 MPa, (**b**) eccentric tableting.

Figure 7 shows the difference between single-tablet compaction and eccentric tableting processes in the tensile strength/bulk density relationship. In the process of single-tablet compaction (Figure 7a), with increasing bulk density of granules the tensile strength of the formed tablets decreased. During this process the same amount of material was weighed, thus the powder-bed-in-die volume decreased with increasing bulk density. The relationship between tensile strength and bulk density was linear, with a high value of the coefficient of determination R^2^ = 0.968. The powder mixture of hydralazine did not fit this linear dependence. Van den Baan and Goodwin [44] came to the conclusion that granules with a higher filling density during continuous compaction of the same mass cause a lower tablet strength, which is in line with our finding.

**Figure 8:**
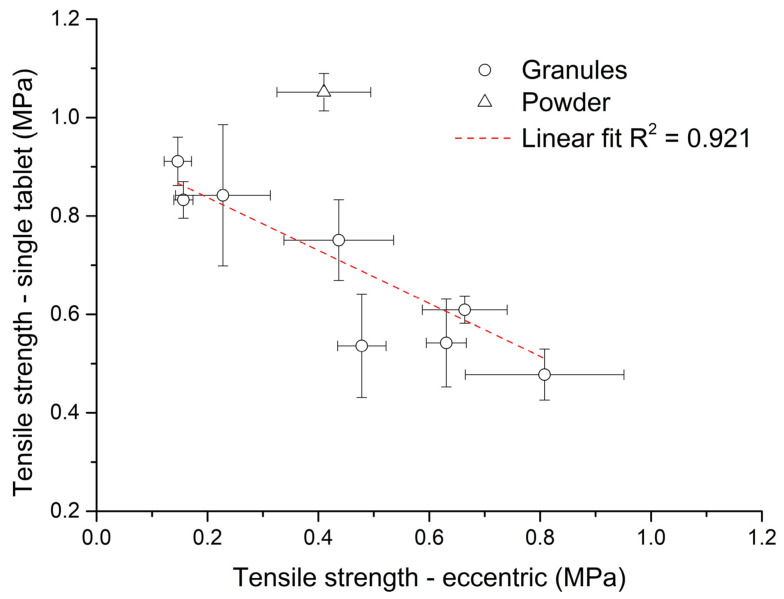
Dependence between tensile strength in single-tablet compaction at 150 MPa and in eccentric tableting.

On the other hand, during the eccentric tableting process (Figure 7b), the tensile strength of tablets increased linearly (R^2^ = 0.917) with an increase in bulk density. During this process, the volume of the powder bed in the die was preserved, and thus a larger mass of material was compressed with increased bulk density. This finding corresponds to Figure 6 and applies to both the granules and the powder mixture. The triangle symbol for powder mixture in Figure 7b also divides the graph into two parts. All points to the right of it are tablets formed from granules with a wet massing time of 120 s, and all points to the left are tablets formed from granules without wet massing time. Granules created with a longer wet massing time had higher bulk density values, and thus a larger mass of material was compressed. The highest tensile strength (*TS*_G8_ = 0.81 ± 0.14 MPa) was for tablets made of PVP granules with a wet massing time of 120 s. Tablets from granules formed from PVP formed at the lowest impeller speed without wet massing time had the lowest strength (*TS*_G5_ = 0.15 ± 0.02 MPa). Since these tensile strength values of tablets made from granules during the eccentric tableting process are exactly the opposite of those from single-tablet compaction, it can be said that tablet strength cannot be determined exactly when switching to another type of tableting machine. Figure 8 shows the dependence between tensile strength in single-tablet compaction at 150 MPa and in eccentric tableting. The dependence between these quantities was linear with the value of the coefficient of determination R^2^ = 0.921. The powder mixture did not fit into this linear relationship. In the further continuation of this work, it would be desirable to focus on the analysis of the uniformity of the drug in individual tablets, but also on changes in the structure and stability of the drug caused by the wet granulation process.

## 4. Conclusions

This article dealt with the influence of high-shear wet-granulation-process parameters on tablet tensile strength and compaction behavior of a powder mixture and granules containing hydralazine. High-shear wet granulation parameters such as types of granulation liquid, impeller speed and wet massing times were monitored. Granulation resulted in reduced dustiness, improved compressibility, and narrower particle-size distribution. Compared to the powder mixture, a reduction in compressibility by more than 50% was achieved using the wet granulation process. The powder mixture and eight different types of granules were compacted into tablets and evaluated using the Heckel, Kawakita and Adams analyses. A significant influence of wet massing time on parameters from the Kawakita and Adams analyses was found. For granules created without wet massing time and with an increase in the granulator impeller speed, the value of the Kawakita parameter decreased slightly; conversely, with a wet massing time of 120 s, it increased. The granules created at higher impeller speed and with longer wet massing time had the highest strength. With a wet massing time of 120 s, the granules were less compressible than without wet massing. Wet massing time had an equally significant effect on tablet tensile strength, regardless of the granulation liquid used. The longer wet massing time, which was also reflected in the span values of the formed granules, also partially affected the strength of the formed tablets. Granules formed with the same wet massing time showed the same trends in tabletability graphs. Tablets created using a single-tablet press and an eccentric tablet press showed opposite values of tensile strength. A linear dependence was found between them with the coefficient of determination R^2^ = 0.921. Tablets from granules with a higher bulk density showed linearly (R^2^ = 0.968) lower strength during batch compaction and, conversely, almost linearly (R^2^ = 0.917) higher strength during eccentric tableting. The results of this work may also be useful in the processing of APIs other than hydralazine, but with similar relevant properties such as particle size, density, solubility, chemical stability, and any other factors that might influence their behavior during granulation.

## Figures and Tables

**Table 1:** Parameters of individual wet granulation experiments. The abbreviation H_2_O in this case refers to distilled water, while PVP is here a 3% aqueous solution of polyvinylpyrrolidone. Wet massing time, W/O, means that granules were created without the wet massing phase.

Experiment	Impeller Speed (rpm)	Wet Massing Time (s)	Granulation Liquid (-)
G1	500	W/O	H_2_O
G2	700	W/O	H_2_O
G3	500	120	H_2_O
G4	700	120	H_2_O
G5	500	W/O	PVP
G6	700	W/O	PVP
G7	500	120	PVP
G8	700	120	PVP

**Table 2:** Properties of the created granules. Abbreviation *d*_mean_—mean granule size, *CI*—compressibility index, *AoR*—angle of repose, *CPS*—compressibility from FT4, N/A mean not assessed.

Experiment	*d*_mean_ (mm)	Span (-)	Sphericity (-)	Bulk Density (g/mL)	*CI* (%)	*AoR* (°)	*CPS* (%)
G1	0.92	1.57	0.89	0.606	12.00	33.43 ± 0.40	2.84 ± 0.27
G2	1.32	1.14	0.87	0.573	11.25	32.19 ± 0.66	2.95 ± 0.33
G3	1.23	0.87	0.87	0.658	10.11	31.62 ± 0.61	2.94 ± 0.29
G4	1.30	0.83	0.87	0.666	11.00	29.36 ± 0.73	2.97 ± 0.29
G5	1.12	1.49	0.86	0.516	15.00	31.59 ± 0.33	3.06 ± 0.28
G6	1.23	1.35	0.86	0.535	12.00	32.14 ± 0.62	3.01 ± 0.34
G7	1.43	0.91	0.87	0.681	8.00	32.46 ± 0.80	2.57 ± 0.29
G8	1.37	0.86	0.86	0.693	11.00	31.36 ± 0.71	2.76 ± 0.23
Powder mixture	N/A	2.98	N/A	0.654	16.30	35.64 ± 0.26	6.24 ± 0.24

**Table 3:** Parameters relating to compaction process. Abbreviations: *P*_y_—Heckel yield pressure, *P*_k_—Kawakita parameter, τ_0_—Adams parameter, UoM—uniformity of mass.

Experiment	*P*_y_ (MPa)	*P*_k_ (MPa)	τ_0_ (MPa)	UoM (%)
G1	141.35 ± 10.65	12.62 ± 0.89	7.01 ± 0.99	1.81
G2	144.33 ± 4.71	11.77 ± 0.18	6.18 ± 0.27	5.35
G3	141.90 ± 5.23	15.19 ± 1.25	10.56 ± 1.70	1.94
G4	145.03 ± 3.82	15.71 ± 0.27	11.65 ± 0.37	3.30
G5	136.89 ± 3.86	10.59 ± 0.68	4.53 ± 0.69	3.58
G6	144.56 ± 3.85	10.49 ± 0.99	4.51 ± 1.14	4.97
G7	145.75 ± 4.61	14.86 ± 1.54	10.22 ± 2.39	4.04
G8	153.96 ± 8.64	17.15 ± 1.02	13.45 ± 1.62	1.96
Powder mixture	108.67 ± 7.56	16.20 ± 0.09	11.94 ± 0.49	1.39

## Data Availability

The data presented in this study are available on request from the corresponding author. The data are not publicly available due to the extensive quantity of values.

## References

[1] Genedy S., Khames A., Hussein A., Sarhan H. (2018). Hydralazine HCl rapidly disintegrating sublingual tablets: Simple dosage form of higher bioavailability and enhanced clinical efficacy for potential rapid control on hypertensive preeclampsia. Drug Des. Devel. Ther..

[2] Agarwal D.S.P., Ahuja A. (1997). Preparation and evaluation of muco-adhesive buccal tablets of hydralazine hydrochloride. Indian J. Pharm. Sci..

[3] Tam S.W., Sabolinski M.L., Worcel M., Packer M., Cohn J.N. (2007). Lack of bioequivalence between different formulations of isosorbide dinitrate and hydralazine and the fixed-dose combination of isosorbide dinitrate/hydralazine: The V-HeFT paradox. Clin. Pharmacokinet..

[4] Matsui F., Robertson D.L., Lovering E.G. (1983). Determination of hydrazine in pharmaceuticals III: Hydralazine and isoniazid using glc. J. Pharm. Sci..

[5] Thong B.Y.H., Tan T.C. (2011). Epidemiology and risk factors for drug allergy. Br. J. Clin. Pharmacol..

[6] Persson A.S., Pazesh S., Alderborn G. (2022). Tabletability and compactibility of α-lactose monohydrate powders of different particle size. I. Experimental comparison. Pharm. Dev. Technol..

[7] Singh B., Pahuja S., Kapil R., Ahuja N. (2009). Formulation development of oral controlled release tablets of hydralazine: Optimization of drug release and bioadhesive characteristics. Acta Pharm..

[8] Al-Suwayeh A.A., El-Shaboury M.H., Al-Baraki S.M., Elgorashy A.S., Taha E.I. (2009). In vitro and in vivo evaluation of sustained release hydralazine hydrochloride tablets prepared by thermal granulation technique. Aust. J. Basic Appl. Sci..

[9] Mughal M.A., Saripella K.K., Kouba C., Iqbal Z., Neau S.H. (2013). Coated hydralazine hydrochloride beads for sustained release after oral administration. Drug Dev. Ind. Pharm..

[10] Vanitha K., Varma M., Ramesh A. (2013). Floating tablets of hydralazine hydrochloride: Optimization and evaluation. Braz. J. Pharm. Sci..

[11] Acharya H., Patel R. (2016). Development and Optimization of Gastro-Retentive Formulation of Hydralazine HCl. Int. J. Pharm. Sci. Drug Res..

[12] Hagelstein V., Gerhart M., Wagner K.G. (2018). Tricalcium citrate–a new brittle tableting excipient for direct compression and dry granulation with enormous hardness yield. Drug Dev. Ind. Pharm..

[13] Persson A.S., Alderborn G. (2018). A hybrid approach to predict the relationship between tablet tensile strength and compaction pressure using analytical powder compression. Eur. J. Pharm. Biopharm..

[14] Vreeman G., Sun C.C. (2022). A powder tabletability equation. Powder Technol..

[15] Adolfsson Å., Nyström C. (1996). Tablet strength, porosity, elasticity and solid state structure of tablets compressed at high loads. Int. J. Pharm..

[16] Wünsch I., Friesen I., Puckhaber D., Schlegel T., Finke J.H. (2020). Scaling tableting processes from compaction simulator to rotary presses—Mind the sub-processes. Pharmaceutics.

[17] Juban A., Briançon S., Puel F., Hoc T., Nouguier-Lehon C. (2017). Experimental study of tensile strength of pharmaceutical tablets: Effect of the diluent nature and compression pressure. EPJ Web Conf..

[18] Berkenkemper S., Kleinebudde P. (2022). Compressibility analysis as robust in-die compression analysis for describing tableting behaviour. RPS Pharm. Pharmacol. Rep..

[19] Yu D., Seelam R.R., Zhang F., Byrn S.R., Hoag S.W. (2021). Evaluation of tableting performance of Poly (ethylene oxide) in abuse-deterrent formulations using compaction simulation studies. J. Pharm. Sci..

[20] Wünsch I., Finke J.H., John E., Juhnke M., Kwade A. (2019). A mathematical approach to consider solid compressibility in the compression of pharmaceutical powders. Pharmaceutics.

[21] Vreeman G., Sun C.C. (2021). Mean yield pressure from the in-die Heckel analysis is a reliable plasticity parameter. Int. J. Pharm. X.

[22] Luo Q., Zhang Q., Wang P. (2022). Hydrochlorothiazide/Losartan Potassium Tablet Prepared by Direct Compression. Pharmaceutics.

[23] Hassanpour A., Ghadiri M. (2004). Distinct element analysis and experimental evaluation of the Heckel analysis of bulk powder compression. Powder Technol..

[24] Nordström J., Welch K., Frenning G., Alderborn G. (2008). On the physical interpretation of the Kawakita and Adams parameters derived from confined compression of granular solids. Powder Technol..

[25] Wang Y., Cao J., Zhao X., Liang Z., Qiao Y., Luo G., Xu B. (2022). Using a Material Library to Understand the Change of Tabletability by High Shear Wet Granulation. Pharmaceutics.

[26] Wang L., Zhao L., Hong Y., Shen L., Lin X. (2023). Attribute transmission and effects of diluents and granulation liquids on granule properties and tablet quality for high shear wet granulation and tableting process. Int. J. Pharm..

[27] Council of Europe (2020). EDQM—European Directorate for the Quality of Medicines. European Pharmacopoeia.

[28] Dudhat S.M., Kettler C.N., Dave R.H. (2017). To Study Capping or Lamination Tendency of Tablets Through Evaluation of Powder Rheological Properties and Tablet Mechanical Properties of Directly Compressible Blends. AAPS PharmSciTech.

[29] Tran D.T., Komínová P., Kulaviak L., Zámostný P. (2021). Evaluation of multifunctional magnesium aluminosilicate materials as novel family of glidants in solid dosage products. Int. J. Pharm..

[30] Macho O., Kabát J., Gabrišová Ľ., Peciar P., Juriga M., Fekete R., Galbavá P., Blaško J., Peciar M. (2018). Dimensionless criteria as a tool for creation of a model for predicting the size of granules in high-shear granulation. Part. Sci. Technol..

[31] Peciar P., Macho O., Peciar M., Fekete R. (2017). Multifunctional Granulator. European Patent.

[32] You Y., Guo J., Li G., Lv X., Wu S., Li Y., Yang R. (2021). Investigation the iron ore fine granulation effects and particle adhesion behavior in a horizontal high-shear granulator. Powder Technol..

[33] You Y., Guo J., Li G., Zheng Z., Li Y., Yu Y., Lv X. (2022). Investigation on the granulation behavior of iron ore fine in a horizontal high-shear granulator. Particuology.

[34] Monaco D., Reynolds G.K., Tajarobi P., Litster J.D., Salman A.D. (2023). Modelling the effect of L/S ratio and granule moisture content on the compaction properties in continuous manufacturing. Int. J. Pharm..

[35] Oka S., Smrčka D., Kataria A., Emady H., Muzzio F., Štěpánek F., Ramachandran R. (2017). Analysis of the origins of content non-uniformity in high-shear wet granulation. Int. J. Pharm..

[36] (2006). Particle Size Analysis—Imange Analysis Methods—Dynamic Image Analysis.

[37] (2008). Representation of Results of Particle Size Analysis—Descriptive and Quantitative Representation of Particle Shape and Morphology.

[38] Fell J.T., Newton J.M. (1970). Determination of tablet strength by the diametral-compression test. J. Pharm. Sci..

[39] Heckel R.W. (1961). Density-Pressure Relationships in Powder Compaction. Trans. Metall. Soc. AIME.

[40] Kawakita K., Ludde K.H. (1971). Some considerations on powder compression equations. Powder Technol..

[41] Adams M.J., McKeown R. (1996). Micromechanical analyses of the pressure-volume relationships for powders under confined uniaxial compression. Powder Technol..

[42] Maroof M.A., Mahboubi A., Noorzad A., Safi Y. (2020). A new approach to particle shape classification of granular mate. Transp. Geotech..

[43] Patel S., Kaushal A.M., Bansal A.K. (2007). Effect of particle size and compression force on compaction behavior and derived mathematical parameters of compressibility. Pharm. Res..

[44] Van den Ban S., Goodwin D.J. (2017). The Impact of Granule Density on Tabletting and Pharmaceutical Product Performance. Pharm. Res..

